# Supplementary material for: Patterns of Intron Gain and Loss in Fungi
Source: PLoS Biol. 2004 Nov 30;2(12):e422. doi: 10.1371/journal.pbio.0020422 (PMC532390; doi:10.1371/journal.pbio.0020422)
Supplement: Table S1 — Also available at http://genes.mit.edu/NielsenEtAl/. (4.3 MB ZIP). [file pbio.0020422.st001.zip › NielsenEtAl/html/199.html]

AN2275.1.NCU00413.1.MG06269.1.FG06931.1


```
 CLUSTAL W (1.82) Multiple Sequence Alignments - Introns Inserted


Sequence 1: NCU00413.1	90 aa
Sequence 2: FG06931.1	90 aa
Sequence 3: MG06269.1	90 aa
Sequence 4: AN2275.1	90 aa
Alignment Length: 90 aa
Number Identitical Residues: 87 aa
Alignment Score (without introns) 3294


MG06269.1 	MIGLVAGGGRTDKPLLK1ASRAKHKFAVKRNSWPKTRGVAMNPVDH~PHGG0GNHQHIGK
NCU00413.1	MIGIVAGGGRTDKPLLK1ASRAKHKFAVKRNSWPKTRGVAMNPVDH~PHGG0GNHQHIGK
FG06931.1 	MIGIVAGGGRTDKPLLK1ASRAKHKFAVKRNSWPKTRGVAMNPVDH~PHGG0GNHQHIGK
AN2275.1  	MIGIVAGGGRTDKPLLK1ASRAKHKFAVKRNSWPKTRGVAMNPVDH0PHGG~GNHQHIGK
          	***:************* **************************** **** ********

MG06269.1 	ASTISRYAAQGQKAGLIAARRTGLLRGTQKTKE
NCU00413.1	ASTISRYAAQGQKAGLIAARRTGLLRGTQKTKE
FG06931.1 	ASTISRYAAQGQKAGLIAARRTGLLRGTQKTKE
AN2275.1  	ASTISRYAAHGQKAGLIAARRTGLLRGTQKTKD
          	*********:**********************:
```
